# Ethanolic Extract of *Origanum vulgare* Suppresses *Propionibacterium acnes*-Induced Inflammatory Responses in Human Monocyte and Mouse Ear Edema Models

**DOI:** 10.3390/molecules23081987

**Published:** 2018-08-09

**Authors:** Lu-Te Chuang, Tsung-Hsien Tsai, Tsung-Jung Lien, Wen-Cheng Huang, Jun-Jen Liu, Hsiang Chang, Mei-Ling Chang, Po-Jung Tsai

**Affiliations:** 1 Department of Biotechnology and Pharmaceutical Technology, Yuanpei University of Medical Technology, Hsinchu 300, Taiwan; ltchuang@mail.ypu.edu.tw (L.-T.C.); hchang@mail.ypu.edu.tw (H.C.); 2 Department of Dermatology, School of Medicine, College of Medicine, Taipei Medical University, Taipei 110, Taiwan; thtsai2@yahoo.com.tw; 3 Department of Human Development and Family Studies, National Taiwan Normal University, Taipei 106, Taiwan; sinna1116@gmail.com (T.-J.L.); wencheng7373@gmail.com (W.-C.H.); 4 School of Medical Laboratory Science and Biotechnology, Taipei Medical University, Taipei 110, Taiwan; jliu_96@tmu.edu.tw; 5 Department of Food Science, Nutrition and Nutraceutical Biotechnology, Shih Chien University, Taipei 104, Taiwan; mlchang@g2.usc.edu.tw; 6 Program of Nutritional Science, School of Life Science, National Taiwan Normal University, Taipei 106, Taiwan

**Keywords:** oregano, *Propionibacterium acnes*, anti-inflammatory, NF-κB, TLR2

## Abstract

Acne vulgaris (acne) is a common inflammatory skin disorder, and *Propionibacterium acnes* plays a major role in the development and progression of acne inflammation. Herbs possessing antimicrobial and anti-inflammatory activity have been applied as a medical option for centuries. In this study, we examined the suppressive effect of ethanolic oregano (*Origanum vulgare*) extract on live *P. acnes*-induced in vivo and in vitro inflammation. Following ethanol extraction of oregano leaves, four compounds with strong antioxidant activity, including rosmarinic acid, quercetin, apigenin, and carvacrol, were identified by high-performance liquid chromatography. Using the mouse ear edema model, we demonstrated that ethanol oregano extracts (EOE) significantly suppressed *P. acnes*-induced skin inflammation, as measured by ear thickness (32%) and biopsy weight (37%). In a separate study, using the co-culture of *P. acnes* and human THP-1 monocytes, EOE reduced the production of interleukin (IL)-8, IL-1β and tumor necrosis factor (TNF)-α up to 40%, 37%, and 18%, respectively, as well as the expression of these three pro-inflammatory mediators at the transcriptional level. Furthermore, EOE inhibited the translocation of nuclear factor-kappa B (NF-κB) into the nucleus possibly by inactivating toll-like receptor-2 (TLR2). The suppressive effect of EOE on live *P. acnes*-induced inflammatory responses could be due, in part, to the anti-inflammatory and antioxidant properties, but not the anti-microbial effect of EOE.

## 1. Introduction

Acne vulgaris (acne) is a common and chronic inflammatory skin disease, and the causes of this disorder are strongly associated with particular factors, including androgen-induced increased sebum production, abnormal keratinization, bacterial colonization and inflammation [1]. Although the exact pathophysiology of acne remains unclear, it is well-known that the Gram-positive anaerobic bacterium, *Propionibacterium acnes* plays an important role in the initiation and prolongation of inflammation [2]. In infected sebaceous follicles, *P. acnes* releases lipases, proteases, and hyaluronidases which damage skin tissues [3]. Furthermore, *P. acnes* induces monocytes through the activation of toll-like receptor 2 (TLR2) to secrete pro-inflammatory cytokines, such as tumor necrosis factor (TNF)-α, interleukin (IL)-1β, and IL-8 [4,5]. Among the cytokines that are released, a chemotactic factor IL-8 is pivotal in attracting neutrophils, basophils, and T cells to the pilosebaceous unit, leading to the development of chronic inflammation [6]. Excessive secretion of pro-inflammatory cytokines has been correlated with acne severity in patients with acne [7]. Thus, developing a suitable approach to suppress or reduce *P. acnes*-induced inflammatory responses might be useful for the treatment of acne vulgaris.

Oregano (*Origanum vulgare*), a member of the Labiatae family (Lamiaceae), is an aromatic shrubby herb that is mainly native to the Mediterranean region, and temperate Western and Southwestern Eurasia. For many years, this flavoring herb has been widely used as spices in daily life, as well as traditional medicines worldwide to treat numerous conditions and symptoms, including relieving rheumatic, digestive or respiratory disorders, lowering blood cholesterol and glucose levels, and also for calming menstrual problems and suppressing the progress of certain tumors [8,9]. Recent in vitro and in vivo studies have demonstrated that extracts of *O. vulgare* have anti-oxidative [10], hepatoprotective [11], anti-diabetic, anti-inflammatory, anti-apoptotic [12,13], and anti-microbial properties [14]. Like other aromatic herbs, oregano leaf is also a rich source of numerous phenolic compounds. It is believed that certain of these constituents, such as rosmarinic acid and carvacrol, are capable of exerting antioxidant properties by scavenging reactive oxygen and nitrogen species, which might be highly associated with numerous beneficial effects [9,15,16,17]. In recent in vitro studies, the ethanolic oregano extracts (EOE) exhibited potent reducing power and DPPH (1,1-diphenyl-2-picrylhydrazyl) radical scavenging activities [18,19] and inhibited growth of Gram-negative and Gram-positive bacteria, including *Salmonella typhimurium*, *Escherichia coli*, and *Listeria* spp. [17,19]. Furthermore, EOE exhibited antioxidant and anti-inflammatory effects on lipopolysaccharide (LPS)-stimulated nitric oxide (NO) production in murine macrophages [18], and a protective effect against cyclophosphamide-induced liver toxicity in mice [11]. These findings indicate that ethanol extracts of oregano contain antioxidant, anti-bacterial, and anti-inflammatory substances that might be applicable to the treatment of acne vulgaris.

To this end, the aims of this study were to extract oregano leaves with ethanol and identify major constituents of EOE using high-performance liquid chromatography (HPLC). Next, to determine if EOE would exert in vivo anti-inflammatory effect in a mouse ear edema model. Third, using a co-culture of *P. acnes* and human THP-1 monocytes as a model, we explored the modulatory effects of EOE on cytokine production and possible working mechanisms. This study provides new knowledge regarding how EOE modulated inflammatory responses in vitro and in vivo.

## 2. Results and Discussion

### 2.1. Determination of Phenolic Compounds in EOE by HPLC Analysis

**Figure 1:**
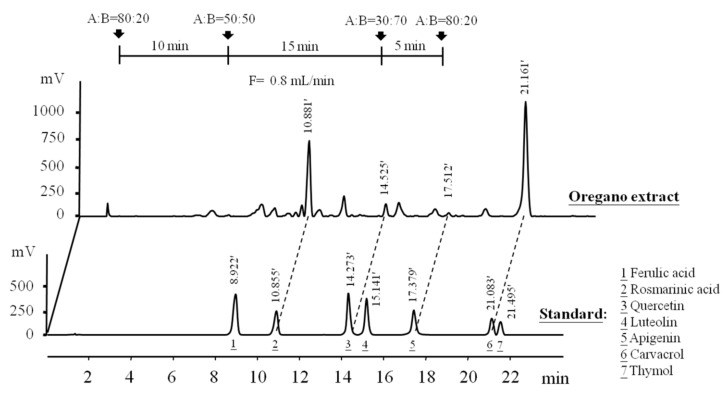
High-performance liquid chromatography (HPLC) chromatograms of phenolic compounds in ethanolic extracts of oregano leaf (EOE). Detection was at 280 nm. Peaks of a mixture known standards are ferulic acid (1), rosmarinic acid (2), quercetin (3), luteolin (4), apigenin (5), carvacrol (6), and (7) thymol. Retention times (min) of phenolic peaks of EOE were shown.

Numerous phenolics from oregano extracts and essential oils had been previously detected and reported [13,20,21]. In this study, we separated and quantified major phenolic constituents of EOE by HPLC; by comparison with the retention times of a mixture of seven authentic standards (Figure 1). The quantities of rosmarinic acid, quercetin, apigenin, and carvacrol in EOE were 15.8 ± 0.89, 3.77 ± 0.48, 1.45 ± 0.25, and 41.0 ± 4.62 mg/g EOE (25.5%, 6.1%, 2.3%, and 66.1% of total identified compounds of EOE), respectively. Another approach by liquid chromatography coupled with mass spectrometry (LC/MS) suggested over 100 compounds from EOE (data unpublished). Recently, Coccimiglio and colleagues [17] reported finding 7 polyphenolic compounds in *Orignum vulgare* from Southern Greece when ethanolic extracts were analyzed using gas chromatography-mass spectrum (GC-MS). When examined and compared two analyses, carvacrol is the only compound identified in both ethanolic extracts. Different extraction and analytical methods might account for the differences between our results and those of Coccimiglio’s [17]. Furthermore, differences in species variety, soil properties, harvest season and other environmental factors (e.g., sunlight, temperature, relative humidity) could account for differences in our findings and theirs regarding the classes and content of various phenolic compounds.

### 2.2. Effect of EOE on P. acnes-Induced Mouse Ear Edema

**Figure 2:**
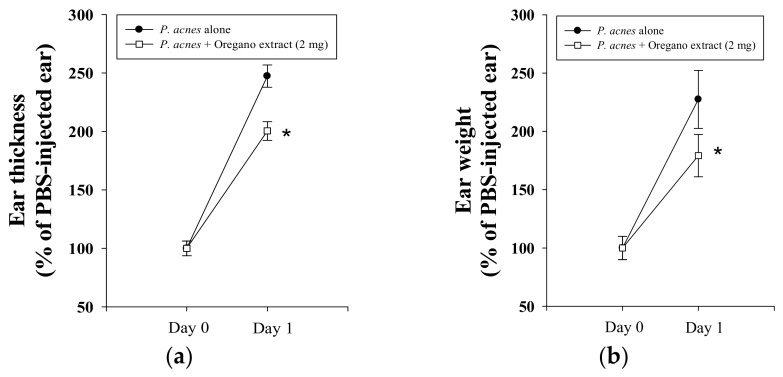
Effects of EOE on *P. acnes*-induced mouse ear edema. The inhibitory effects of EOE on *P. acnes*-induced ear edema in mice were assessed by measuring ear thickness (**a**) and ear biopsy weight (**b**). Each value represents the mean ± SD of three independent experiments. The value with a symbol (*) is significantly different from each other at *p* < 0.05.

The mouse ear edema model is a well-established and reliable in vivo method for screening for the presence and determining the amounts of tumor-promoting, pro-inflammatory, and anti-inflammatory compounds in plant extracts [22]. To investigate the possible in vivo suppressive effect of EOE on *P. acnes*-induced inflammation, the right ear of mice was intradermally injected with a fixed quantity of bacterial culture, at the same site in test animals and controls. After 24 h-incubation, *P. acnes* stimulated mouse ear swelling was estimated by comparing ear thickness (by 2.5-fold) and ear biopsy weight (by 2.3-fold) to the untreated control (Figure 2). Co-injection of mouse ear with EOE significantly reduced *P. acnes*-induced ear edema by 32% and 37%, as estimated by thickness and weight measurements, respectively (Figure 2). These data provide evidence that the topical application of EOE has an in vivo suppressive effect on *P. acnes*-induced inflammation. Additionally, we used human THP-1 monocytes to confirm the anti-inflammatory effect of EOE on *P. acnes*-induced inflammatory responses and to investigate the mechanism underlying the beneficial biological effect of EOE.

### 2.3. Effect of EOE on Cytokine Production

**Figure 3:**
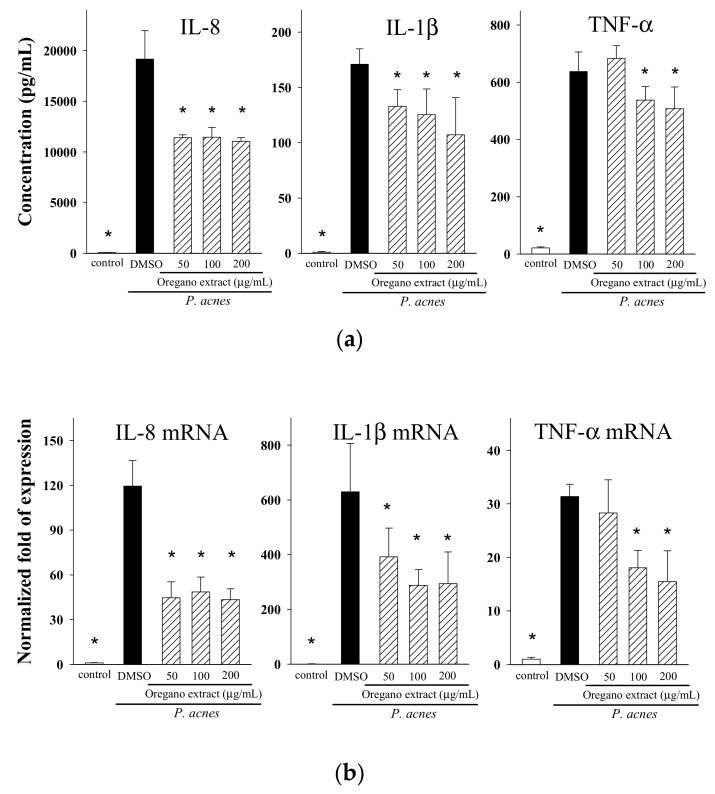
Effects of various concentrations of EOE supplementation on cytokine secretion and mRNA level. Interleukin (IL)-8, IL-1β, and tumor necrosis factor (TNF)-α concentrations (**a**), and their mRNA levels (**b**) by *P. acnes*-stimulated THP-1 cells. Cells were co-cultured with *P. acnes* (200 μg/mL) and different concentrations (50, 100 or 200 μg/mL) of EOE for 24 h. The levels of cytokine mRNA were normalized to glyceraldehydes 3-phosphate dehydrogenase (GAPDH) mRNA and expressed as multiples of change with the control (untreated THP-1 cells). The bars indicate the mean ± SD of three independent experiments. The values with a symbol (*) are significantly different from the DMSO (vehicle) group at *p* < 0.05.

To first determine whether EOE is cytotoxic to human THP-1 monocytes, we cultured cells with different amounts (0, 25, 50, 100 or 200 μg/mL) of EOE for 24 h. Over this range of EOE extract, we found that EOE was not cytotoxic to THP-1 cells (data not shown). Recently, phenolic compounds were shown to reduce the levels of pro-inflammatory mediators in various cell lines [23,24]. To examine whether phenolic compound-containing EOE could also modulate inflammatory responses, THP-1 cells were stimulated with live *P. acnes* and increasing levels (50, 100 or 200 μg/mL) of EOE. The levels of three pro-inflammatory mediators were markedly increased following *P. acnes* simulation (Figure 3a). However, increasing amounts of EOE reduced the concentrations of Interleukin (IL)-8, IL-1β, and tumor necrosis factor (TNF)-α in the culture medium up to 40%, 37%, and 18%, respectively. We also inquired if EOE affected the expression of these same three mediators at the transcriptional level. As shown in Figure 3b, EOE significantly decreased the mRNA levels of IL-8 and IL-1β. Likewise, the level of TNF-α mRNA was lowered when the EOE concentration was 100 μg/mL or higher.

Several studies have demonstrated that oregano extracts inhibit the growth of certain types of bacteria [14,17,19]. In this study, although we have documented that EOE appears to exert its anti-inflammatory properties by lowering cytokine production, we were concerned that the decrease in pro-inflammatory mediator production was not due to the anti-inflammatory properties of EOE, but instead to the anti-bacterial effect of EOE. In fact, results of our broth microdilution antibacterial assay showed that the minimal inhibitory concentration (MIC) and minimal bactericidal concentration (MBC) of EOE were 8 and 16 mg/mL, respectively; thus, both the MIC and MBC values were at least 40-fold higher than the highest concentration (200 μg/mL) of EOE which we supplemented into the THP-1 cell culture. We conclude, therefore, that the anti-microbial properties of EOE probably do not account for the lowering of *P. acnes*-induced pro-inflammatory cytokine production by EOE.

We further investigated whether major phenolic constituents of EOE play critical roles in the modulation of inflammatory responses. Results from our previous studies indicated that doses of rosmarinic acid in the 2.5 to 10 μM range significantly suppressed the production of IL-8, but had no such suppressive effect on the production of IL-1β or TNF-β [25]. Similarly, IL-8 production was also reduced by incubation of THP-1 cells with quercetin (2.5, 5 or 10 μM) or apigenin (25 or 50 μM) [26]. However, supplementation of THP-1 cells with carvacrol (up to 200 μM) did not affect the levels of *P. acnes*-induced IL-8 (data not shown). We estimated that one milliliter of EOE (200 µg/mL) contains 3.2 µg of rosmarinic acid (equivalent to 8.9 µM), 0.8 µg of quercetin (equivalent to 2.6 µM), 0.3 µg of apigenin (equivalent to 1.1 µM), and 8.0 µg of carvacrol (equivalent to 53.3 µM). Of these four major polyphenolic components in the EOE, the concentrations of rosmarinic acid and quercetin are greater than the minimally effective concentration of each of the respective phenolic compounds (>2.5 µM) mentioned above. Thus, rosmarinic acid, quercetin, and other unidentified components, but not apigenin and carvacrol, in EOE might account for the suppressive effect of the oregano extract on IL-8 production in the co-culture of *P. acnes* and THP-1 monocyte model.

### 2.4. Effect of EOE on NF-κB Activation and TLR-2 Expression

**Figure 4:**
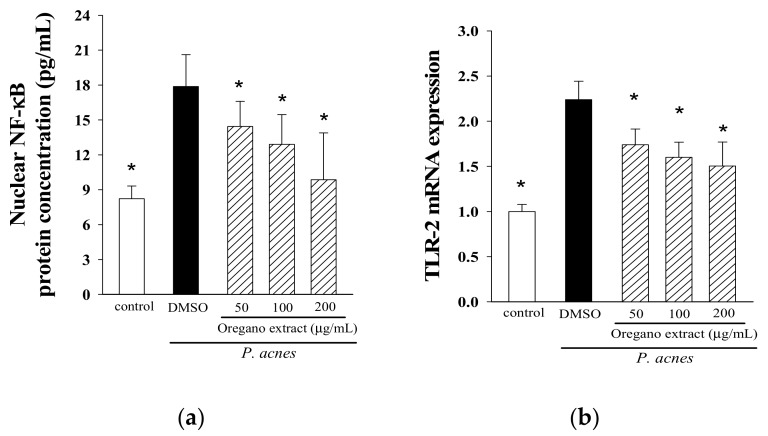
Effects of EOE supplementation on activation of nuclear factor kappa-B (NF-κB) (**a**) and mRNA expression of toll-like receptor-2 (TLR2) (**b**). THP-1 cells were co-incubated with *P. acnes* and different concentrations (50, 100 or 200 μg/mL) of EOE for 16 h. The TLR2 mRNA expression was normalized to glyceraldehydes 3-phosphate dehydrogenase (GAPDH) mRNA and expressed as multiples of change with the control. The bars indicate the mean ± SD of three independent experiments. The values with a symbol (*) are significantly different from the DMSO (vehicle) group at *p* < 0.05.

We also wanted to know if the modulation of NF-κB translocation and TLR2 expression by EOE might play critical roles in the suppression of pro-inflammatory cytokine production by THP-1 cells. The results in Figure 4 show that the levels of translocated NF-κB into the nucleus and TLR2 mRNA were increased in response to *P. acnes* stimulation as compared to the non-stimulated control. However, incubation of THP-1 monocytes with different concentrations (50, 100 or 200 μg/mL) of EOE significantly suppressed the activation of NF-B p65 and over-expression of TLR2 by up to 43% and 33%, respectively. These results indicate that co-incubation of a *P. acnes* culture and monocyte cells with EOE suppress TLR2-mediated NF-κB translocation into the nucleus of THP-1 monocytes.

To date, details of the molecular mechanism underlying *P. acnes*-associated skin inflammation remain unclear because the pathological process involves a complex interplay between abnormal proliferation and function of skin cells and immune microenvironments induced by a series of bacterial and biochemical events. For example, *P. acnes* produces propionate and other chemicals that have cytotoxic properties [27] and stimulate the innate immune response through the microbial-sensing receptors, such as TLR2 and TLR4 [4] to initiate the mitogen-activated protein kinases (MAPK)/NF-κB signaling [25,28]. It is well-known that MAPK and NF-κB signalings are responsible for the over-expression of numerous genes of pro-inflammatory mediators, subsequently leading to the influence of immune and inflammatory responses. In this study, we reported that EOE significantly suppressed *P. acnes*-stimulated inflammatory responses in THP-1 cells. Our findings are in accord with results of previous studies showing that phenolic compound-rich extracts from rosemary, clove, bitter melon, and other plants lower pro-inflammatory cytokine production through the suppression of TLR2-mediated MAPK/NF-κB signaling [24,25,26].

The formation of free radicals, such as the reactive oxygen species (ROS) (such as hydrogen peroxide, superoxide, hydroxyl radical) and reactive nitrogen species (RNS) (such as nitric oxide, peroxynitrite) are increased during the process of inflammation. Oxidative stress caused by the excess or inappropriately controlled free radicals has been postulated to play a critical role in prolonging the *P. acnes*-mediated inflammatory state [29]. For example, using a murine macrophage cell model, Tsai and coworkers demonstrated that over-expression of inflammatory inducible nitric oxide synthase (iNOS) and type 2-cyclooxygenase (COX-2) by *P. acnes* involved ROS-dependent NF-κB and transcription factor AP-1 activation [30]. Pastore and colleagues [31] also demonstrated that the generation of ROS and RNS is highly associated with the triggering of transcription factor AP-1/NF-κB Signaling. These findings suggested that free radicals generated from the inflammatory process would re-initiate and amplify inflammation, and lead to chronic inflammation and inflammation-related diseases.

In addition to their anti-inflammatory properties, the flavonoids and phenolic compounds in herbal extracts inhibit in vitro antioxidant production, at least in part, by chelating ferrous ions and scavenging free 2,2′-azino-bis(3-ethylbenzothiazoline-6-sulphonic acid) (ABTS), 2,2-diphenyl-1-picrylhydrazyl (DPPH) or nitric oxide (NO) radicals in numerous studies [32,33]. The antioxidant potential of natural compounds also has been reported in several studies [34]. In the present study, the four constituents we identified in EOE extracts (rosmarinic acid, quercetin, apigenin, and carvacrol) have recently been shown to exert potent antioxidant activities in murine B16 melanoma cells, human HepG2 cells, human Caco-2 cells, and mice [23,32,35,36]. Furthermore, based on the fact that rosmarinic acid acts as a potent free radical scavenger and suppresses gene expression, and production of pro-inflammatory mediators, Adomako-Bonsu and colleagues [32] have proposed that the potential protective and therapeutic benefits of rosmarinic acid or other phenolics on topical diseases/disorders were likely the result of the combined effects of anti-inflammatory and antioxidant properties. Thus, our results indicate that the suppressive effect of phenolic-rich EOE on *P. acnes*-induced inflammatory responses in mouse ear edema and THP-1 cell models might be through the inactivation of TLR2-mediated NF-κB cell signaling and down-regulation of downstream pro-inflammatory mediator production, the result of combined both the anti-inflammatory and antioxidant properties.

The results of this study demonstrate that EOE exerts potent anti-inflammatory effects by suppressing *P. acnes*-induced inflammation, and it is conceivable that EOE could be applied as one of the functional ingredients in medical or cosmetic products to relieve acne or other skin disorders. However, one of the limitations of this application is that suppressive effect of EOE may be varied from batch to batch. This is because oregano leaves obtained worldwide are different in species variety and the classes and content of phenolic compounds. One of the options is to apply the single identified polyphenol from EOE, such as rosmarinic acid, quercetin into the medical or facial applications. In future application of EOE, the high cost of isolation, purification, etc. might not be favorable for industrial production of medical or facial products. Moreover, the synergetic effects of EOE on the reduction of mouse ear edema might disappear when utilizing any single individual phenolic compound. Furthermore, although EOE suppressed *P. acnes*-induced inflammatory responses by intradermal injection, we need a suitable delivery system to ensure EOE could be successfully delivered into skin after topical administration. Future in vivo investigations involving in formulation design on the anti-inflammatory effects of EOE-containing products on inflammation are required.

Recently, Nakatsuji and colleagues [37] reported that synergistic effect of Christie, Atkins, Munch-Peterson (CAMP) factor from *P. acnes*, and acid sphingomyelinase (ASMase) from the host cells play one of the major roles in the involvement of *P. acnes* virulence, leading to the elevated acne inflammation. They further demonstrated that the *P. acnes*-induced in vitro and in vivo inflammation could be suppressed by the inhibition of both live *P. acnes*-induced expression of bacterial CAMP factor and host ASMase [37]. Based on the result that *P. acnes* CAMP factor and cellular ASMase can be only simultaneously detected when live *P. acnes* was co-incubated with mammalian cells or intradermally injected into mouse ears, we suggest that the similar over-expression pattern of both proteins might be involved in the process of the inflammation in the current study. Even this study did not conclude whether suppressive effect of EOE on *P. acnes*-stimulated inflammation is due, in part, to the inhibition of expression of both *P. acnes* CAMP factor and host ASMase. The investigation on the relationship between EOE (or other herbal extracts, chemicals, etc.) and live *P. acnes*-stimulated inflammation is certainly critical and could be a new approach to investigate and develop potential anti-*P. acnes* inflammatory agents.

In summary, we have separated, identified and quantified the amounts of four major phenolic constituents in EOE using HPLC analysis. This phenolic-rich EOE relieves ear swelling in a *P. acnes*-induced mouse ear edema model and exerts its anti-inflammatory effects by suppressing production and over-expression of pro-inflammatory IL-8, IL-1β, and TNF-α, at least in part, by suppressing TLR2-mediated NF-κB signaling in human monocytes. The suppressive effect of EOE on *P. acnes*-induced inflammatory responses might be due to the combined effects of anti-inflammatory and antioxidant potentials, but not to anti-bacterial properties. Thus, EOE is a potential anti-inflammatory and antioxidant agent for the medical application in inflammatory acnes.

## 3. Materials and Methods

### 3.1. Chemicals

3-(4,5-dimethylthiazol-2-yl)-2,5-diphenyltetrazolium bromide (MTT), apigenin, carvacrol, dimethylsulfoxide (DMSO), ferulic acid, glucose, luteolin, quercetin, and thymol were purchased from Sigma Chemical Co. (St. Louis, MO, USA). Rosmarinic acid was purchased from Extrasynthese (Genay, France). Brain heart infusion (BHI) broth was from Difco (Detroit, MI, USA). Phosphate-buffered saline (PBS), RPMI 1640 medium, fetal bovine serum (FBS), penicillin and streptomycin were obtained from Gibco (Carlsbad, CA, USA). The ELISA assay kits for detecting IL-8, IL-1β, and TNF-α were purchased from Invitrogen (Carlsbad, CA, USA). The NF-κB/p65 ActivELISA kit was obtained from Imgenex (San Diego, CA, USA). All reagent-grade organic solvents were from Burdick and Jackson (Muskegon, MI, USA).

### 3.2. Ethanol Extraction of Oregano Leaves

Dried oregano leaves were from Tomax Enterprise Co. (Taipei, Taiwan). Oregano leaves were extracted based on the modified method of Tsai et al. [25]. Briefly, 10 g of finely ground oregano powders were immersed and extracted with 100 mL of ethanol and stirred continuously at room temperature for 4 h. After the first extraction, the residue was re-extracted overnight with 100 mL of ethanol. The ethanolic extract was then centrifuged (12,000× *g*) for 10 min, and the combined ethanol filtrates were collected and taken to dryness in a rotary evaporator. The dried extract was weighed and re-constituted with a known volume of DMSO to a concentration of 400 mg/mL.

### 3.3. Characterization of Phenolic Compounds in EOE

Contents of particular phenolic compounds were determined by HPLC with a Phenomenex C-18 reversed phase silica column (300 mm × 3.9 mm; i.d., 10 m; Phenomenex, Torrance, CA, USA), HPLC pumps (Ecom LCP 4100, Praha, Czech Republic) and a UV detector (Ecom LCD 2084, Praha, Czech Republic). Chromatographic analysis was carried out using the Peak-ABC Chromatography Data Handling System (E–Chrom Tech Co., Ltd., Taipei, Taiwan). The mobile phase contained solvent A (acetonitrile/water/acetic acid, 15:80:0.85) and solvent B (methanol), with a linear gradient starting with A/B (80:20) with a steady flow rate of 0.8 mL/min, changing to A/B (50:50) over a period of 10 min, then to A/B (50:50) for 15 min, and to A/B (80:20) for 5 min. The effluent was monitored at 284 nm to identify most polyphenols. A mixture of reference authentic standards were used to identify compounds in the ethanolic extract of oregano. Peak identification was based on HPLC retention times compared with those of selected standards and confirmed by spiked samples with standard materials.

### 3.4. Bacterial and Cell Cultures and Growth Conditions

The strain of *P. acnes* (BCRC10723) and human monocytic THP-1 cell line (BCRC 60430) were obtained from the Bioresource Collection and Research Center (Hsinchu, Taiwan). *P. acnes* was incubated in BHI broth with 1% (*w*/*v*) glucose in an anaerobic the BBL GasPak system (Becton Dickinson, Cockeysville, MD, USA). THP-1 cells were maintained in RPMI 1640 medium supplemented with 10% (*v*/*v*) heat-inactivated FBS, penicillin (100 U/mL), and streptomycin (100 μg/mL) in a 5% CO_2_ fully humidified environment at 37 °C. To monitor cell viability, the methods of MTT and AlamarBlue^®^ (Invitrogen, Carlsbad, CA, USA) assays were used.

### 3.5. Effect of EOE on P. acnes-Induced Inflammation In Vivo

Eight-week-old male ICR (Institute of Cancer Research) mice were from Animal Center of College of Medicine, National Taiwan University (Taipei, Taiwan), and housed in the laboratory animal facility at National Taiwan Normal University according to an animal use protocol approved by the Institutional Animal Care and Use Committee (IACUC Approval No. LAC10301). Mice were randomly divided into two groups (5 per group) and fed a standard chow diet (LabDiet, St. Louis, MO, USA) and water ad libitum. Using the modified Nakatsuji’s method described elsewhere [38], we determined in vivo anti-inflammatory effects of EOE in a *P. acnes*-induced mouse ear edema model. *P. acnes* (6 × 10^7^ CFU per 10 μL in PBS) was inoculated into the right ear of ICR mice by intradermally injection, and left ears (the control) received an equal amount (10 μL) of PBS. After *P. acnes* or PBS injection, EOE (2 mg/10 μL) in 5% DMSO in PBS (*v*/*v*) or vehicle (5% DMSO in PBS) was injected into the same site of both ears. Preliminary studies have shown that this dose of EOE (2 mg) is optimal for topical testing without causing visible skin irritation (data not shown). Twenty-four hours after *P. acnes* injection, the increase in thickness of mouse ear swelling was measured using a micro-caliper (Mitutoyo, Kanagawa, Japan). All experimental mice were then sacrificed by carbon dioxide asphyxiation. Each 4-mm ear punch biopsy was immediately weighed. The degree of edema in each mouse was evaluated respectively, based on results of the thickness and weight difference between both ears. The increase in ear thickness and weight of the *P. acnes*-injected ear was expressed as a percentage of the PBS-injected control.

### 3.6. Determination of Cytokine Production by THP-1 Cells

The cellular assay models using live *P. acnes* infected with mammalian cells, including HaCaT keratinocytes, human peripheral blood mononuclear cell-derived monocytes, osteoblasts and THP-1 monocytes, have been established [37,39,40,41]. Using a co-culture model of *P. acnes* and human THP-1 monocytes as we previously described [41], we determined the modulatory effects of EOE on *P. acnes*-stimulated cytokine production. A batch of *P. acnes* was cultured to reach the log phase, and bacterial pellets were harvested at 10,000× *g* for 5 min, rinsed with PBS for three times, resuspended in RPMI medium, and for subsequent infection with THP-1 cells. Human THP-1 cells were seeded at a density of 1 × 10^6^ cells/mL per well with FBS-free medium in a 24-well plates, and then stimulated with medium containing only live *P. acnes* (wet weight 200 μg/mL of bacteria; 7.5 × 10^7^ colony-forming unit/mL) as the control, or *P. acnes* and different concentrations of EOE (50, 100 or 200 μg/mL) in a 5% CO_2_ fully humidified environment at 37 °C. After incubation for 24 h, the cell-free supernatants were collected, and the concentrations of TNF-α, IL-1β, and IL-8 were determined with the aid of respective commercial enzyme immunoassay kits.

### 3.7. Analysis of mRNA Levels by Real-Time Reverse Transcription Polymerase Chain Reaction

Total RNA of THP-1 cells was extracted and isolated with TRIzol reagent (Invitrogen; Carlsbad, CA, USA), and cDNA was synthesized from 2 μg of RNA in a reaction mixture of oligo (dT) primers and reverse transcriptase (Promega, Madison, WI, USA). Real-time polymerase chain reaction (PCR) analysis was performed using an iCycler iQ Real-Time detection system (Bio-Rad, Hercules, CA, USA). To amplify cDNA, primer sets for target genes (IL-1β, IL-8, TNF-α, TLR2, and glyceraldehydes 3-phosphate dehydrogenase (GAPDH)) and thermal cycling conditions were used for all PCR assays as described elsewhere [25]. The relative amounts of the PCR products were measured and analyzed using iQ™5 optical system software (ver. 2.1; Bio-Rad). The mRNA levels of each sample that were analyzed for all genes of interest were normalized to that of the GAPDH mRNA.

### 3.8. Determination of Nuclear Translocation of NF-κB p65

To determine if activation of nuclear transcription factor (NF-κB) was modulated by EOE, THP-1 monocytes (3 × 10^6^ cells/mL) were incubated with serum-free medium containing only 200 μg/mL live *P. acnes*, or in combination with different concentrations of EOE (50, 100 or 200 μg/mL) for 16 h, respectively. The NF-κB/p65 ActivELISA™ kit was used to determine the cytosolic p65 subunit translocation into the nucleus [25].

### 3.9. Statistical Analyses

Data were analyzed by Student’s *t*-test by using SPSS software (SPSS for windows 17.0; SPSS Inc., Chicago, IL, USA) to determine differences between means of the DMSO vehicle and oregano treatments. Means differences were considered significant at the *p* < 0.05 levels.
